# Glucagon-like peptide-1 derived cardioprotection does not utilize a KATP-channel dependent pathway: mechanistic insights from human supply and demand ischemia studies

**DOI:** 10.1186/s12933-016-0416-3

**Published:** 2016-07-19

**Authors:** Joel P. Giblett, Richard G. Axell, Paul A. White, Sophie J. Clarke, Liam McCormick, Philip A. Read, Johannes Reinhold, Adam J. Brown, Michael O’Sullivan, Nick E. J. West, David P. Dutka, Stephen P. Hoole

**Affiliations:** Department of Interventional Cardiology, Papworth Hospital, Papworth Everard, Cambridge, CB23 3RE UK; Department of Cardiovascular Medicine, University of Cambridge, Cambridge, UK; Department of Clinical Engineering, Addenbrooke’s Hospital, Cambridge, UK

**Keywords:** Ischemia–reperfusion injury, Glucagon-like peptide-1, Cardioprotection, KATP

## Abstract

**Background:**

Glucagon-like peptide-1 (7–36) amide (GLP-1) protects against stunning and cumulative left ventricular dysfunction in humans. The mechanism remains uncertain but GLP-1 may act by opening mitochondrial K-ATP channels in a similar fashion to ischemic conditioning. We investigated whether blockade of K-ATP channels with glibenclamide abrogated the protective effect of GLP-1 in humans.

**Methods:**

Thirty-two non-diabetic patients awaiting stenting of the left anterior descending artery (LAD) were allocated into 4 groups (control, glibenclamide, GLP-1, and GLP-1 + glibenclamide). Glibenclamide was given orally prior to the procedure. A left ventricular conductance catheter recorded pressure–volume loops during a 1-min low-pressure balloon occlusion (BO1) of the LAD. GLP-1 or saline was then infused for 30-min followed by a further 1-min balloon occlusion (BO2). In a non-invasive study, 10 non-diabetic patients were randomized to receive two dobutamine stress echocardiograms (DSE) during GLP-1 infusion with or without oral glibenclamide pretreatment.

**Results:**

GLP-1 prevented stunning even with glibenclamide pretreatment; the Δ % dP/dt_max_ 30-min post-BO1 normalized to baseline after GLP-1: 0.3 ± 6.8 % (p = 0.02) and GLP-1 + glibenclamide: −0.8 ± 9.0 % (p = 0.04) compared to control: −11.5 ± 10.0 %. GLP-1 also reduced cumulative stunning after BO2: −12.8 ± 10.5 % (p = 0.02) as did GLP-1 + glibenclamide: −14.9 ± 9.2 % (p = 0.02) compared to control: −25.7 ± 9.6 %. Glibenclamide alone was no different to control. Glibenclamide pretreatment did not affect global or regional systolic function after GLP-1 at peak DSE stress (EF 74.6 ± 6.4 vs. 74.0 ± 8.0, p = 0.76) or recovery (EF 61.9 ± 5.7 vs. 61.4 ± 5.6, p = 0.74).

**Conclusions:**

Glibenclamide pretreatment does not abrogate the protective effect of GLP-1 in human models of non-lethal myocardial ischemia.

*Trial registration* Clinicaltrials.gov Unique Identifier: NCT02128022

**Electronic supplementary material:**

The online version of this article (doi:10.1186/s12933-016-0416-3) contains supplementary material, which is available to authorized users.

## Background

Percutaneous coronary intervention (PCI) is the cornerstone of treatment for acute myocardial infarction (AMI) but ischemia–reperfusion (IR) injury may account for up to 50 % of the final infarct size [1]. Adjunctive cardioprotective strategies to limit or prevent IR injury are desperately needed [2]. One strategy is ischemic conditioning (IC) in which repeated episodes of brief ischemia applied to a tissue protect against larger ischemic insults both locally and in distant tissues [3–5]. IC mediates cardioprotection through the opening of the mitochondrial K-ATP (mK-ATP) channel and closure of the mitochondrial permeability transition pore (mPTP) [6]. Blockade of mK-ATP with the sulfonylurea glibenclamide (glyburide) has been shown to abolish IC in humans [7, 8]. Sulfonylureas are important medications for the treatment of diabetes mellitus but they may cause an increase in cardiovascular mortality, perhaps as a result of interfering with protection from ischemic conditioning [9].

IC has proved challenging to translate into patient benefit so other strategies have focused on pharmacological agents to limit IR injury [2]. One promising agent is Glucagon-like peptide-1 (7–36) amide (GLP-1), an incretin hormone with an insulinotropic action, released from the gut in response to a food bolus [10]. Exogenous GLP-1 receptor agonists and drugs that inhibit its breakdown are used to treat type 2 diabetes mellitus. A beneficial off-target effect of GLP-1 is protection against IR injury [11–13]. GLP-1 abolishes PCI-induced left ventricular (LV) stunning (supply ischemia) [14, 15] and improves LV function during dobutamine stress echocardiography (demand ischemia) [16]. Evidence from animal studies suggests that GLP-1 may act through similar subcellular signaling pathways to IC [17, 18]. We sought to establish whether blockade of mK-ATP would abolish the cardioprotection conferred by GLP-1 in human studies of both demand and supply ischemia.

## Methods

### Study population

Patients were aged over 18 years old and able to give informed consent. They had proximal left anterior descending artery (LAD) disease requiring PCI (supply protocol) or any coronary artery disease requiring revascularization (demand protocol). Patients were not enrolled in both studies. Exclusion criteria were presence of diabetes mellitus, use of nicorandil or ranolazine, a serum creatinine >200 μmol/l, presence of a cardiac pacemaker, valvular heart disease, impaired left ventricular systolic function, recent myocardial infarction or life expectancy less than 6-months.

### Ethics, consent and permissions

Both protocols were approved by the local ethics committee (14/EE/0056 and 08/H0304/68) and informed, written consent was obtained from all participants before enrolment. Studies were carried out in accordance with institutional guidelines. The studies were registered on clinicaltrials.gov (NCT02128022) and ISRCTN (ISRCTN69686930) and were performed in accordance with the Declaration of Helsinki.

### Supply ischemia protocol

**Fig. 1:**
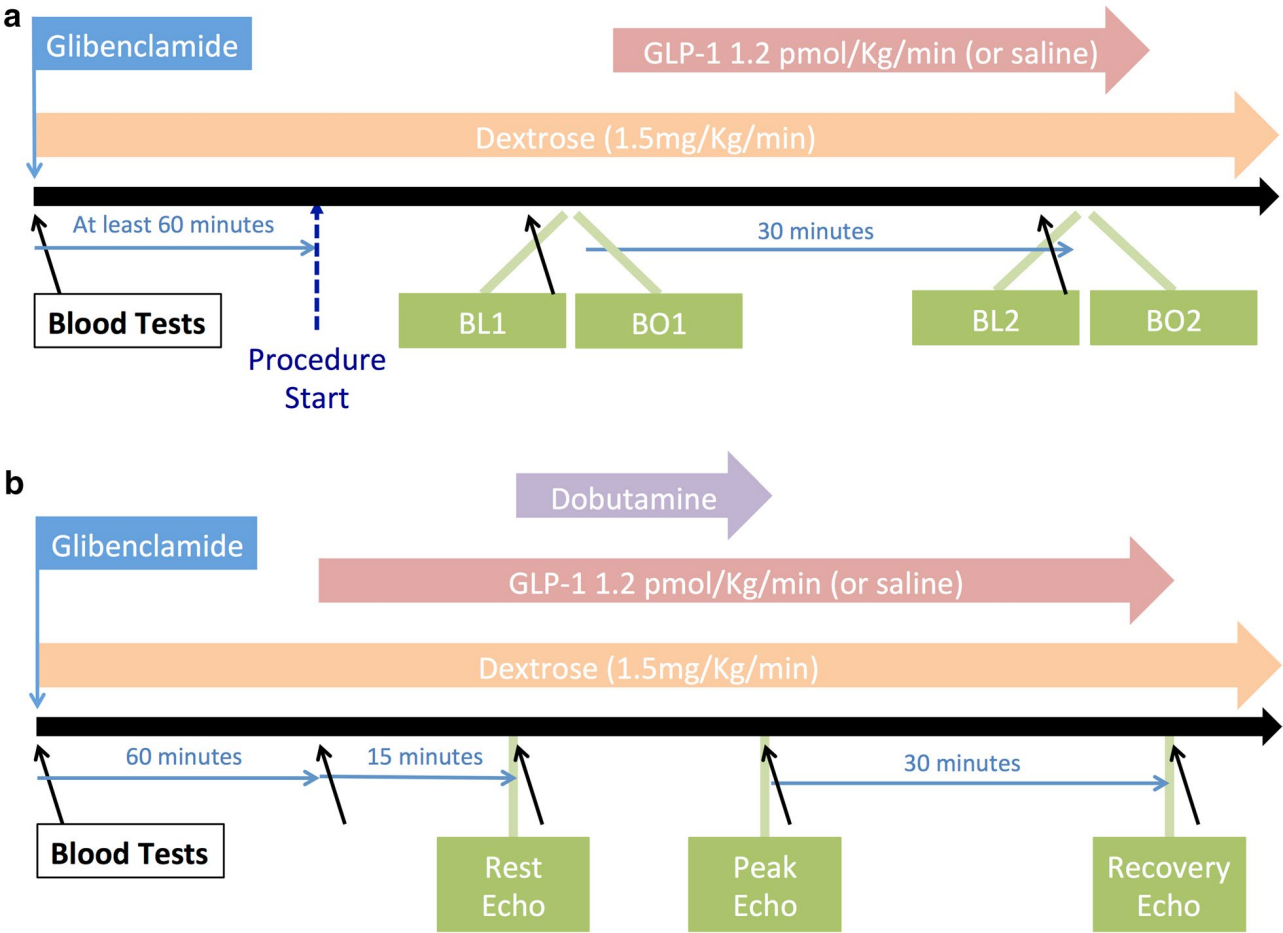
Structure of supply and demand protocols. **a** Timeline for the supply ischemia protocol. Blood tests were taken immediately before the two balloon occlusions. GLP-1 was administered until BO2 was complete. **b** The study timeline for the demand ischemia protocol

A timeline for this protocol is shown in Fig. 1. Patients were fasted for 6 h prior to the procedure and received standard dual antiplatelet loading with aspirin and clopidogrel. Patients were allocated to one of four groups: saline control, GLP-1 alone, saline with glibenclamide and GLP-1 with glibenclamide. Patients received glibenclamide 5 mg orally at least 60 min prior to their PCI procedure and had a dextrose 20 % infusion initiated at 1.5 mg/kg/min to minimize the risk of hypoglycemia. In mechanistic studies an oral dose of 5 mg was sufficient to inhibit IC in humans [19].

**Fig. 2:**
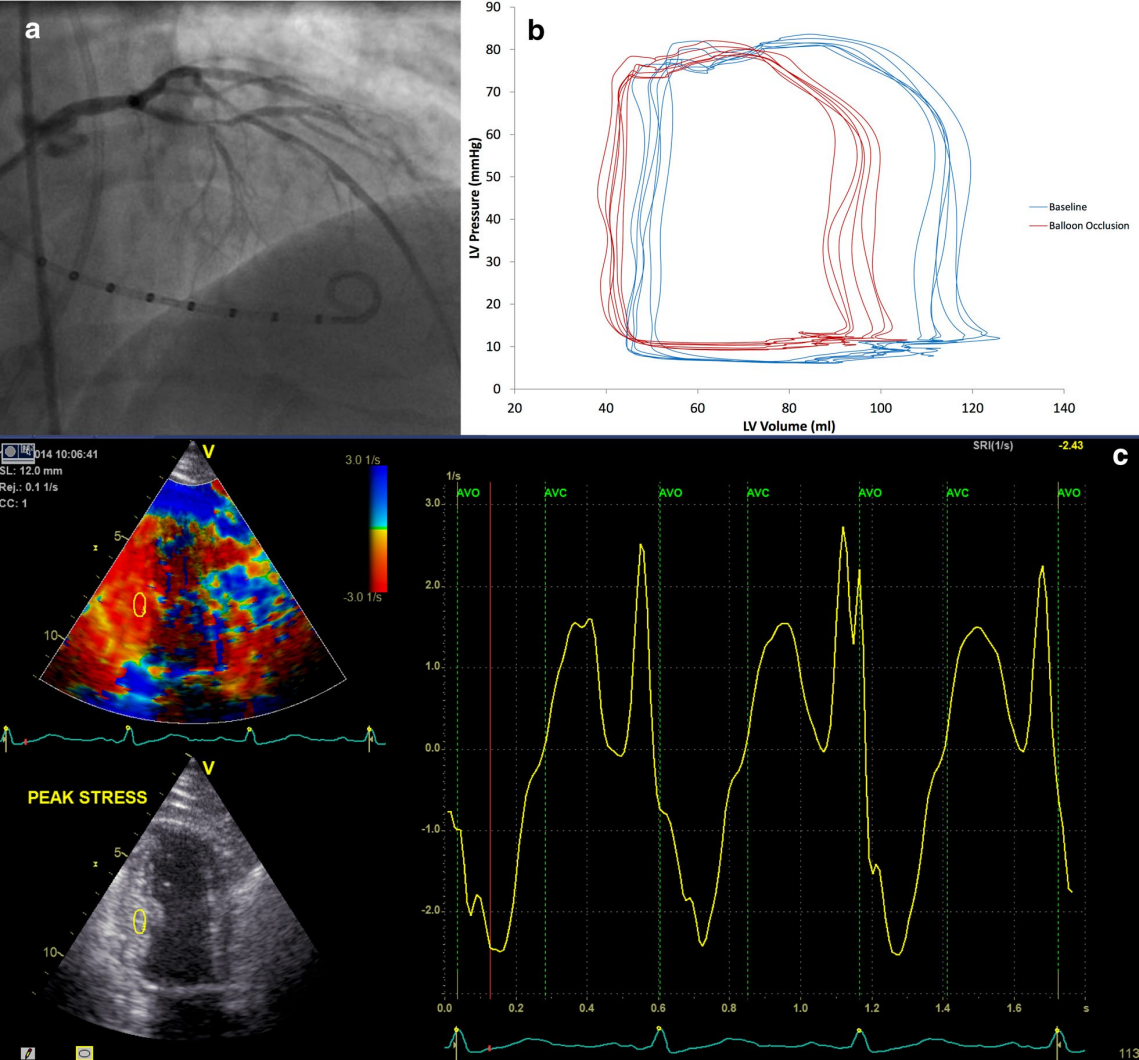
Examples of methodology. **a** Conductance catheter in position in the left ventricle during an injection of contrast into the left coronary artery. The proximal LAD lesion can be seen. **b** Pressure–volume loops generated at baseline (*blue*) and during occlusion of the LAD (*red*). **c** Dobutamine stress echo at peak stress. Strain rate is shown to the *right* with the peak systolic strain rate the most negative point between aortic valve opening and closure

Sheaths were placed in the right femoral artery, right radial artery and right femoral vein. Between 70 and 100 U/kg of unfractionated heparin was administered to maintain an activated coagulation time of >250 s throughout the procedure. An 8-electrode 7F conductance catheter (Millar Instruments, Houston, USA) was connected to an MPVS Ultra (Millar Instruments, Houston, USA) signal-conditioning unit in series with an ADInstruments PowerLab 16/30 Series (ADInstruments, New South Wales, Australia) 16-channel amplifier. It was positioned along the longitudinal ventricular axis with the tip at the apex of the LV (Fig. 2). The conductance catheter was calibrated and corrected for parallel conductance [14, 20] as described in Additional file 1.

The left coronary ostium was intubated with a guide catheter and the lesion was crossed with a coronary guide wire. Baseline pressure–volume loops were recorded from the conductance catheter after the lesion was crossed (BL1) during a breath-hold in mid-expiration. Similarly, pressure–volume loops were recorded at the end of a 1-min low-pressure (<4 atmospheres) coronary balloon inflation (BO1) within the lesion. Contrast was injected during balloon inflation to confirm coronary occlusion.

Patients randomized to receive GLP-1 then received an intravenous infusion of GLP-1 at 1.2 pmol/kg/min started immediately after balloon deflation. This dose was selected as it had been effective in inducing cardioprotection in previous studies, with minimal side-effects [14]. Those patients in the control and glibenclamide only groups received an infusion of normal saline at the same rate. After 30-min, further pressure–volume loops were collected (BL2) followed by recordings after a further 1-min balloon occlusion (BO2). Patients then received conventional coronary angioplasty and stenting of the lesion at the discretion of the treating physician. One-minute balloon occlusions are long enough to induce ischemic dysfunction and stunning of the myocardium but will not activate the protective effect of ischemic conditioning [21].

### Pressure–volume loop analysis

Conductance catheter data analysis was performed offline using LabChart (AdInstruments, New South Wales, Australia), by investigators blinded to treatment allocation. Five cardiac cycles recorded at each time-point (BL1, BO1, BL2, BO2) were sampled (Fig. 2) and used to determine systolic parameters: dP/dt_max_ (maximum rate of isovolumic pressure increase), stroke volume and ejection fraction and diastolic parameters: dP/dt_min_ (maximum rate of isovolumic pressure decline) and Tau (time constant of isovolumic pressure decline). Tau is calculated from the conductance catheter-derived P_t_ (time constant of pressure relaxation) which is measured from the time of peak rate of pressure decline (dP/dt_min_) to 5 mmHg above end-diastolic pressure.

### Demand ischemia protocol

Patients underwent two dobutamine stress echocardiograms (DSE) in randomly allocated order, performed at least 1 week apart. Patients attended in the morning following an overnight fast and received a GLP-1 infusion with or without oral glibenclamide 5 mg administered 60-min prior to the GLP-1 infusion. GLP-1 was started at 1.2 pmol/kg/min followed by dobutamine administration 15 min later. An intravenous infusion of 20 % dextrose started at 1.5 mg/kg/min was given during both DSE to minimize the risk of glibenclamide-induced hypoglycaemia. Each patient acted as their own control. Intravenous dobutamine infusion was incrementally increased from 10 μg/kg/min to 20, 30 and 40 μg/kg/min at 3-min intervals. Up to 2 mg of intravenous atropine could be given if necessary to reach the target heart rate. Both GLP-1 and dextrose infusions continued throughout the protocol until recovery was complete (Fig. 1). Criteria for stopping included, achievement of target heart rate (220 – Age * 0.85 BPM), angina, ischemic ECG changes (ST depression or elevation greater than 2 mm), rise in systolic blood pressure to >240 mmHg, fall in systolic blood pressure to <100 mmHg or severe arrhythmia.

### Transthoracic echocardiographic analysis

Two-dimensional transthoracic tissue Doppler imaging (TDI) was performed in the left recumbent position at rest, peak stress and 30-min recovery. Images were recorded in 2, 3 and 4 chamber apical views allowing a 12-segment model (apical and mid segments) of regional wall motion to be assessed. Segments were classified as ischemic or non-ischemic according to whether or not they were subtended by a coronary stenosis >70 % by Quantitative Coronary Angiographic (QCA) analysis, as validated by Baptista et al. [22]. Relevant ischemic-segments were analyzed offline to determine the systolic tissue velocity (Vs), strain index (SI), and strain rate index (SRI) by a clinician blinded to treatment allocation (Fig. 2). Global left ventricular function was calculated by ejection fraction (EF) using Simpson’s Biplane method and mitral annular systolic velocity (MASV) averaged from six sites. Inter-observer variability and intra-observer variability were assessed using images from three randomly selected patients. This was expressed as an Interclass Correlation Coefficient (ICC) with 95 % confidence interval [23].

### Biochemistry

During the supply ischemia protocol venous blood was sampled at baseline and immediately prior to BO1 and BO2. In the demand ischemia protocol venous blood was sampled prior to starting dextrose, GLP-1 and dobutamine infusions, at peak stress and at 30-minute recovery (Fig. 1).

GLP-1 (7–36) amide, free fatty acid, insulin and glucose levels were assayed. Samples for GLP-1 were drawn into tubes containing dipeptidyl peptidase 4 (DPP4) inhibitor (Millipore, UK) to prevent degradation and measured using a commercially available assay (Meso Scale Discovery, MD, USA). Glucose samples were measured by YSI 2300 STAT Plus analyzer (YSI Life Sciences, UK).

### Statistical analysis

In the supply protocol a power calculation was carried out based on previous work by our group [14]. Analysis of non-diabetic patients who had previously received saline or GLP-1 infusion after a balloon occlusion in a similar protocol to this study had a delta dP/dt_max_ at BL2 of −13.4 ± 9.3 % and −1.4 ± 8.5 % respectively. We estimated that to detect a difference of 8 % at BL2 (α = 0.05, β = 0.2) a sample size of 8 per group was required.

In the demand ischemia protocol, previous data has shown that a GLP-1 infusion caused an additional increase in ejection fraction at peak stress from 70.8 ± 4.96 to 77.0 ± 4.39 % in a paired study [16]. We estimated that to detect a 5 % change in ejection fraction a sample size of 7 patients were needed (α = 0.05, β = 0.2).

All analyses were pre-specified. Continuous data are presented as mean ± SD unless otherwise stated. Paired and unpaired Student’s t test or Mann–Whitney U test were used to compare continuous variables as appropriate, and Chi Square test was used for categorical variables. In the supply ischemia study, 4-way comparisons between groups were carried out with a one-way independent ANOVA. A p value of <0.05 was deemed statistically significant.

## Results

### Supply ischemia protocol

Thirty-three patients were recruited into the 4 groups detailed above (Additional file 1: Figure S1). In one case the operator was unable to cross the aortic valve with the conductance catheter and this patient was excluded. Demographic details for the groups are summarized in Additional file 1: Table S1 and confirm that they were well matched.

Biochemistry data is summarized in Table 1. Levels of plasma GLP-1 (7–36) amide were below 3.0 pg/ml at baseline and during BO1 confirming no significant difference between groups. Plasma levels of GLP-1 (7–36) amide rose at BO2 in groups treated with GLP-1 compared with those administered only saline, with no significant difference between the GLP-1-treated groups (p = 0.41). Plasma levels of insulin were significantly elevated at BO2 in patients treated with glibenclamide and the combination of GLP-1 and glibenclamide caused an even greater rise although the response was highly variable between individuals. Patients treated with glibenclamide also had significant suppression of free fatty acids at BO1 and BO2. Plasma glucose levels were higher at BO2 in glibenclamide treated groups, compared with control, despite the rise in insulin levels, reflecting the co-administration of dextrose alongside glibenclamide.

**Table 1:** Metabolic changes during supply ischemia protocol

	Baseline		Balloon occlusion 1 (BO1)		Balloon occlusion 2 (BO2)	
		p value*		p value*		p value*
GLP-1 (7–36) amide, pg/ml						
Control	2.9 ± 3.57	–	3.6 ± 4.13	–	2.9 ± 1.84	–
GLP-1 only	2.8 ± 1.43	0.51	2.5 ± 2.04	0.49	87.5 ± 37.1	<0.001
Glibenclamide only	1.9 ± 1.66	0.46	1.1 ± 0.77	0.11	1.3 ± 0.54	0.07
GLP-1 glibenclamide	2.2 ± 0.59	0.57	2.3 ± 1.32	0.37	78.9 ± 31.1	<0.001
Insulin, pmol/L						
Control	79 ± 26	–	59 ± 29	–	58 ± 29	–
GLP-1 only	77 ± 52	0.91	63 ± 85	0.82	95 ± 64	0.16
Glibenclamide only	103 ± 93	0.51	292 ± 245	0.02	382 ± 297	0.02
GLP-1 glibenclamide	90 ± 51	0.63	412 ± 244	<0.01	1133 ± 1089	0.03
Glucose, mmol/l						
Control	5.1 ± 0.3	–	5.3 ± 0.3	–	5.4 ± 0.4	–
GLP-1 only	5.0 ± 0.3	0.83	5.0 ± 0.3	0.51	4.7 ± 0.3	0.20
Glibenclamide only	5.3 ± 0.3	0.66	6.3 ± 0.4	0.06	6.8 ± 0.4	0.02
GLP-1 glibenclamide	5.5 ± 0.3	0.44	5.9 ± 0.4	0.27	5.9 ± 0.7	0.45
Free fatty acids, μmol/L						
Control	371 ± 364	–	1399 ± 973	–	1050 ± 637	–
GLP-1 only	473 ± 367	0.57	1213 ± 897	0.69	917 ± 516	0.64
Glibenclamide only	358 ± 201	0.93	633 ± 434	0.06	439 ± 248	0.02
GLP-1 glibenclamide	356 ± 183	0.91	846 ± 490	0.16	503 ± 307	0.04
Dextrose infusion, mg kg^−1^ min^−1^						
Glibenclamide only	–	–	1.69 ± 0.53	–	1.69 ± 0.53	–
GLP-1 glibenclamide	–	–	2.25 ± 1.6	–	2.44 ± 1.59	–

* p value compared with control group

There were no significant differences between the baseline hemodynamic parameters of groups (Additional file 1: Table S2). Table 2 describes how parameters of systolic and diastolic function change during the supply ischemia protocol for each group. All groups suffered ischemic LV dysfunction during BO1 compared to BL1, with a significant deterioration in all measured parameters of systolic and diastolic function. There was no significant difference between any groups at BO1. At BL2 there was recovery of dP/dt_max_ and dP/dt_min_ to BL1 levels in both GLP-1 treated groups. There was impaired recovery of dP/dt_max_ and dP/dt_min_ in control and glibenclamide only groups with significant stunning compared with BL1. Glibenclamide had a neutral effect on systolic and diastolic function during balloon occlusion and recovery.

**Table 2:** Change in parameters of systolic and diastolic function (compared to BL1) in supply ischemia protocol

	Baseline 1 (BL1)	Balloon occlusion 1 (BO1)		Baseline 2 (BL2)		Balloon occlusion 2 (BO2)	
	%	Δ %	p value*	Δ %	p value*	Δ %	p value*
dP/dt_max_							
Control	100	−15.4 ± 13.6	0.012	−11.5 ± 10.0	<0.01	−25.7 ± 9.6	<0.001
GLP-1 only	100	−16.1 ± 10.2	<0.01	−0.3 ± 6.8	0.92	−12.8 ± 10.5	0.011
Glibenclamide only	100	−16.1 ± 14.4	0.011	−10.0 ± 4.7	<0.01	−21.7 ± 10.5	<0.01
GLP-1 glibenclamide	100	−13.5 ± 9.7	<0.01	−0.8 ± 9.0	0.82	−14.9 ± 9.2	<0.01
Ejection fraction							
Control	100	−18.3 ± 14.1	0.01	−13.8 ± 15.5	0.03	−31.1 ± 16.0	<0.001
GLP-1 only	100	−17.9 ± 13.3	0.01	−8.4 ± 12.7	0.10	−11.7 ± 17.8	0.10
Glibenclamide only	100	−17.2 ± 13.4	0.01	−12.7 ± 19.6	0.14	−26.9 ± 14.2	<0.001
GLP-1 glibenclamide	100	−16.9 ± 13.8	0.01	−8.4 ± 4.8	<0.01	−13.1 ± 9.5	<0.01
Stroke volume							
Control	100	−19.6 ± 18.1	0.03	−16.4 ± 16.3	0.02	−31.2 ± 16.6	<0.001
GLP-1 only	100	−19.8 ± 17.5	0.01	−5.8 ± 27.1	0.56	−12.6 ± 28.8	0.26
Glibenclamide only	100	−16.8 ± 17.8	0.08	−12.6 ± 18.2	0.12	−23.2 ± 22.1	0.04
GLP-1 glibenclamide	100	−14.8 ± 11.1	0.01	−0.2 ± 7.3	0.96	−5.6 ± 4.4	0.03
dP/dt_min_							
Control	100	−25.1 ± 14.7	<0.01	−10.9 ± 4.7	<0.001	−33.3 ± 8.6	<0.001
GLP-1 only	100	−21.3 ± 8.3	<0.001	+5.0 ± 11.6	0.26	−21.3 ± 8.7	0.04
Glibenclamide only	100	−26.4 ± 14.3	<0.001	−7.1 ± 12.5	0.17	−28.6 ± 13.1	<0.001
GLP-1 glibenclamide	100	−19.6 ± 6.1	<0.001	+2.9 ± 13.0	0.55	−14.3 ± 13.6	0.02
Tau							
Control	100	+34.7 ± 16.9	<0.01	+12.7 ± 7.8	<0.01	+46.4 ± 15.4	<0.001
GLP-1 only	100	+27.3 ± 11.6	<0.001	+8.0 ± 18.6	0.26	+31.0 ± 36.7	0.05
Glibenclamide only	100	+30.5 ± 25.8	<0.01	+13.1 ± 19.9	0.14	+36.2 ± 26.3	<0.001
GLP-1 glibenclamide	100	+31.3 ± 27.2	<0.001	+5.5 ± 10.2	0.17	32.5 ± 13.2	<0.001

* p value compared to BL1 for each group

**Fig. 3:**
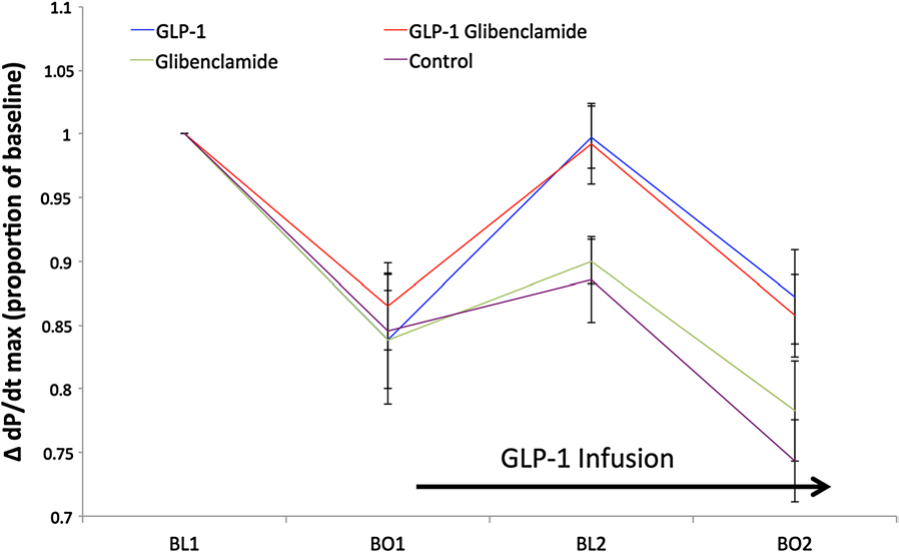
Systolic function (ΔdP/dt_max_) in supply ischemia protocol. Mean ± SEM. There were no statistically significant differences between groups at BO1. At BL2, dP/dt_max_ for GLP-1 was significantly improved compared to control (p = 0.02) and compared to glibenclamide alone (p < 0.01). This difference was maintained for comparison with control at BO2 (p = 0.02) and was numerically better than glibenclamide at BO2 (p = 0.12). dP/dt_max_ for GLP-1 + glibenclamide was significantly improved at BL2 compared to control (p = 0.02) and glibenclamide alone (p = 0.03). Again this difference was maintained for comparison with control at BO2 (p = 0.02) and trended towards improvement for glibenclamide alone at BO2 (p = 0.14). There was no statistically significant difference between GLP-1 treated groups, nor any difference between the control and glibenclamide only groups, at any timepoint

**Fig. 4:**
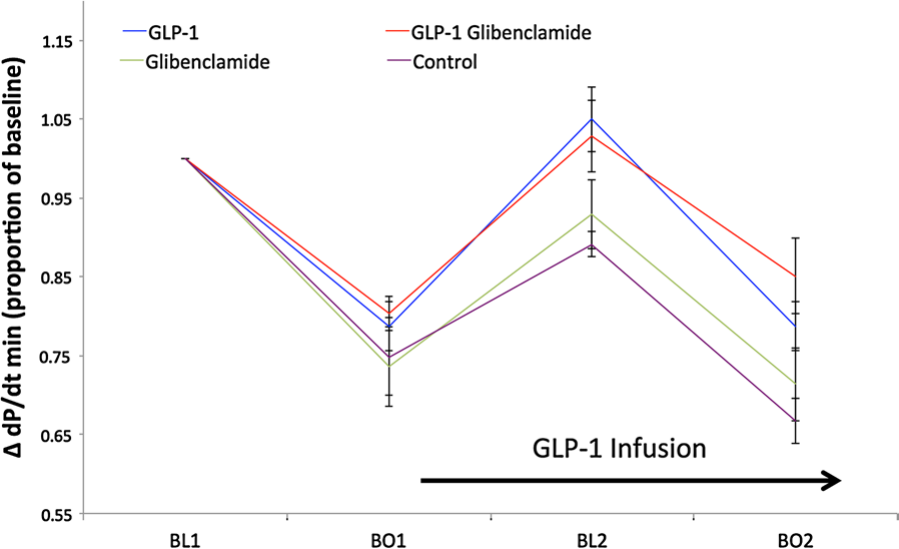
Diastolic function (ΔdP/dt_min_) in supply ischemia protocol. Mean ± SEM. There were no statistically significant differences between groups at BO1. At BL2, dP/dt_min_ for GLP-1 was significantly improved compared to control (p < 0.01) and numerically better than glibenclamide alone (p = 0.07). The difference between GLP-1 vs. control was maintained at BO2 (p = 0.01). At BL2, dP/dt_min_ for GLP-1 + glibenclamide was again significantly improved compared to control (p < 0.01), and numerically better than glibenclamide alone (p = 0.15). These differences persisted for BO2 (p < 0.01 and 0.06 respectively). There was no statistically significant difference between GLP-1 treated groups, nor any difference between the control and glibenclamide only groups, at any timepoint

Cumulative stunning after the second balloon occlusion was only observed in groups not receiving GLP-1; systolic and diastolic function did not deteriorate below the level observed at BO1 in GLP-1 treated groups. Change in dP/dt_max_ (Fig. 3) and dP/dt_min_ (Fig. 4) are shown for each group at the specified time points. GLP-1 infusion also improved EF at BL2 and BO2 compared to control although this was only statistically significant at BO2. Tau appeared to improve with GLP-1 but this was not statistically significant. There was no significant change in heart rate (p = 0.14) or mean arterial pressure (p = 0.67) between balloon occlusions, or between individual groups.

### Demand ischemia protocol

Ten patients were recruited into the demand ischemia protocol. Two patients did not go on to have both DSE (one patient withdrew and one needed urgent revascularization prior to second DSE). Detailed demographic data are summarized in the Additional file 1: Table S3.

Overall there were no significant differences in hemodynamic parameters between the DSE. Importantly, GLP-1 infusion did not increase heart rate during the 15 min prior to the DSE in the GLP-1 only DSE (57 ± 3.8 vs. 58 ± 3.9, p = 0.44). There was no significant difference in the RPP at peak stress between the DSE. These data are summarized in Additional file 1: Table S4.

There was a rise in GLP-1 levels at peak stress during both DSE of over 50-fold compared with pre-DSE levels. Insulin levels rose in both DSE but were twice as high for the GLP-1 + glibenclamide group (p = 0.04). There was increased suppression of free fatty acids with GLP + glibenclamide. Biochemistry data for the demand ischemia protocol are summarized in Additional file 1: Table S5.

**Fig. 5:**
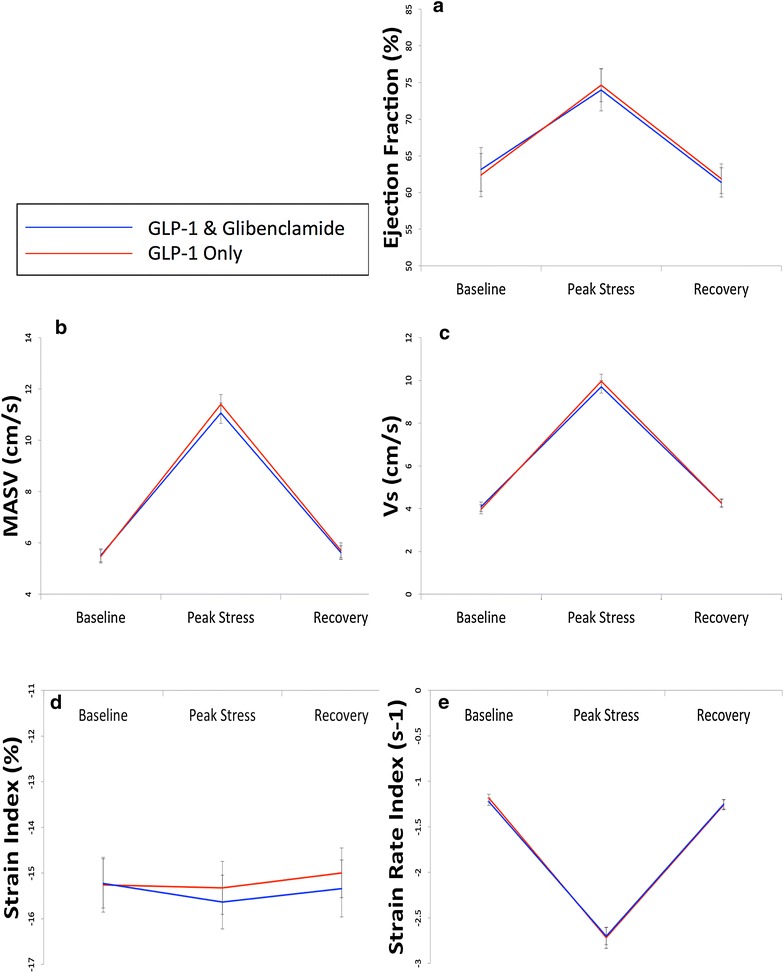
Systolic function in demand ischemia protocol. Mean ± SEM. Changes in global and regional systolic function at baseline, peak stress and 30-min recovery. There are no statistically significant differences between the two studies at any timepoint. **a** Ejection fraction. **b** Mitral annular systolic velocity. **c** Peak systolic tissue velocity (Vs). **d** Peak systolic strain (SI). **e** Peak systolic strain rate (SRI)

There was no difference in global indices of systolic function between the paired DSE at either peak stress or recovery, indicating that glibenclamide did not attenuate the effect of GLP-1 (Table 3). Regional tissue Doppler indices were also unchanged when ischemic segments alone, and all segments together were analyzed (Additional file 1: Table S6). Changes in the global and regional tissue Doppler indices are shown in Fig. 5. The ICC for inter- and intra-observer variability respectively was 0.899 (0.850–0.931) and 0.922 (0.863–0.975) for Vs; 0.802 (0.698–0.873) and 0.831 (0.748–0.886) for SI; 0.817 (0.637–0.908) and 0.844 (0.691–0.901) for SRI.

**Table 3:** Change in parameters of global systolic function, and ischemic regional segments in demand ischemia protocol

	GLP-1 only	GLP-1 glibenclamide	p value
Ejection fraction (%)			
Baseline	62.4 ± 8.4	63.1 ± 8.5	0.45
Peak stress	74.6 ± 6.4	74.0 ± 8.1	0.76
Recovery	61.9 ± 5.7	61.4 ± 5.6	0.74
MASV, cm/s			
Baseline	5.47 ± 1.79	5.51 ± 1.78	0.82
Peak stress	11.40 ± 2.69	11.06 ± 2.80	0.24
Recovery	5.70 ± 1.99	5.62 ± 1.75	0.51
Peak systolic velocity (Vs), cm/s			
Baseline	3.76 ± 1.89	3.92 ± 2.07	0.38
Peak stress	9.86 ± 3.08	9.59 ± 2.81	0.18
Recovery	3.95 ± 1.95	4.04 ± 1.56	0.98
Strain index (SI), %			
Baseline	−15.3 ± 5.83	−15.1 ± 5.30	0.68
Peak stress	−15.5 ± 5.52	−15.6 ± 6.09	0.92
Recovery	−14.8 ± 5.35	−15.3 ± 6.21	0.48
Strain rate index (SRI), s-1			
Baseline	−1.19 ± 0.74	−1.25 ± 0.51	0.29
Peak stress	−2.68 ± 1.69	−2.79 ± 1.17	0.65
Recovery	−1.24 ± 0.92	−1.25 ± 0.69	0.71

## Discussion

In both the supply and demand ischemia protocols, addition of glibenclamide did not affect GLP-1 cardioprotection; GLP-1 protection against both ischemic left ventricular dysfunction and stunning remained. The implication of this study is that GLP-1 cardioprotection is not mediated through mK-ATP dependent pathways. Similar metabolic profiles (between glibenclamide-treated groups) ruled-out a metabolic explanation for the cardioprotection observed. Only the presence or absence of GLP-1 appeared to be associated with a protective effect and this was independent of both mK-ATP blockade and changes in myocardial substrate and insulin levels.

IC acts via mK-ATP-dependent subcellular signaling pathway that is activated by up-stream RISK/SAFE pathways of cardioprotection [11, 18]. IC protection from non-lethal endothelial IR injury has been shown to be K-ATP dependent in humans. This protection is blocked with glibenclamide [8]. Although IC has not been shown to protect against stunning or ischemic dysfunction in humans [24], GLP-1 does protect the LV against this model of non-lethal IR injury, as confirmed in this study. The current study also shows that co-administration of glibenclamide with GLP-1 does not affect GLP-1 cardioprotection. Therefore cardioprotection against non-lethal IR injury by GLP-1 is not mediated through mK-ATP dependent subcellular signaling pathways in humans. This is surprising since a number of other studies suggest K-ATP dependent signaling pathways mediate the actions of GLP-1 on the vasculature. K-ATP channels are involved in GLP-1 dependent relaxation of the aorta in a rat model, and endothelial function in human forearms [17, 25]. Animal studies have shown that phosphoinositol-3 kinase (PI3K) and Akt are consistently activated during treatment with GLP-1 [11, 18, 26]. Our study suggests that either human GLP-1 cardioprotection is mediated through an alternative pathway, or that PI3K-Akt signaling acts through an alternative mechanism to protect against myocardial stunning and ischemic dysfunction. There is considerable cross-talk in the RISK/SAFE pathways and it may be that these kinases are acting on an alternative distal target [27]. Since evidence suggests that the known GLP-1 receptor is not present on the human ventricular cardiomyocyte [28], GLP-1 may act remote to the myocardium or by binding an as yet unidentified receptor.

In the supply protocol both glibenclamide alone and GLP-1 alone produced a rise in insulin levels and a fall in free fatty acids compared to saline control. However, only GLP-1 had a cardioprotective effect. Glibenclamide only produced changes in metabolic parameters without an observed difference in the function of the ventricle compared to control—this suggests that the metabolic effect of glibenclamide does not lead to alteration in cardiac function in this model. The combination of GLP-1 and glibenclamide produced even greater hyperinsulinemia but offered no additional protection beyond that of GLP-1 alone. Insulin levels with glibenclamide were similar to those observed during hyperinsulinemic clamp studies published by our group, which also showed a neutral effect of insulin on myocardial performance during ischemia [29]. It has been suggested that the mechanism for GLP-1 cardioprotection is a switch in cardiomyocyte metabolism towards more energy efficient myocardial glucose utilization [30]. Glibenclamide is insulinotropic through blockade of K-ATP on the pancreatic beta cell. The metabolic environment created with addition of glibenclamide and dextrose (increased availability of glucose, reduced availability of fatty acids and increased cellular uptake of glucose driven by hyperinsulinemia) should promote increased myocardial glucose utilization. However this environment did not result in cardioprotection without GLP-1, nor did it enhance cardioprotection beyond giving GLP-1 alone. These findings are consistent with our earlier human work, which did not detect a change in transmyocardial substrate utilization after GLP-1 infusion [15]. Taken as a whole, it seems unlikely that GLP-1 cardioprotection is mediated by straight-forward metabolic switching toward glucose metabolism or by change in insulin levels.

Studies have suggested that, in the human heart, the GLP-1 receptor is only present in the sinoatrial node [28]. GLP-1 receptor agonists such as Exenatide have been shown to increase heart rate [31]. In this study, the GLP-1 infusion did not affect heart rate. Since GLP-1 did not affect heart rate, or afterload and the peak rate pressure product achieved, there was no evidence from this study that GLP-1 protection was mediated through an effect on the peripheral vasculature reducing cardiac workload. Further work to confirm this is needed.

The mechanism underlying GLP-1 cardioprotection in non-lethal IR injury remains speculative. Animal evidence of mK-ATP dependent protection may still be applicable to lethal IR injury in humans. Alternatively, mK-ATP independent aspects of subcellular pro-survival kinase pathways may be involved in GLP-1 cardioprotection. Studies have suggested that adenosine may mediate some of the observed GLP-1 effects [32]. Adenosine released from remote endothelial cells in response to GLP-1 receptor binding may elicit an inotropic effect by inducing hyperemia, or alternatively by a direct protective effects on cardiomyocytes [33]. Evidence of effects independent of the known GLP-1 receptor pathway have also been described [18, 34, 35] and GLP-1 may activate as yet unknown subcellular signaling pathways to confer protection. The protection offered involved native GLP-1 rather than a stable GLP-1 receptor agonist such as Exenatide. This may mean that breakdown products such as GLP-1 (9–36) amide may be responsible for some of the observed cardioprotection. GLP-1 (9–36) has been implicated in cardioprotection in some animal studies [34, 35]. A number of other protective effects of GLP-1 have been explored. These include effects on platelet aggregation and thrombosis [36], and anti-inflammatory effects [37]. Whilst these effects may be important in protection against, and recovery from myocardial infarction, they are unlikely to explain the protection conferred against myocardial stunning.

## Limitations

The study does not present direct evidence of blockade of the cardiomyocyte mK-ATP channel. This was assumed on the basis of previous studies [19]. It may also be inferred in this study as the insulinotropic effect of glibenclamide on the pancreatic beta cell (which was seen in the biochemical results) is dependent on sarcoplasmic K-ATP blockade [38]. This insulinotropic effect of glibenclamide necessitated the co-administration of dextrose to prevent hypoglycemia. Whilst the analyses suggest that this has not affected our conclusions, it is not possible to completely exclude some confounding effect.

The supply ischemia protocol did not measure load-independent indices of ventricular function such as the end-systolic pressure volume relationship (Ees) due to the difficulty of simultaneously recording a family of pressure–volume loops during preload reduction (IVC balloon occlusion) whilst also balloon occluding the coronary artery. However, blood pressure and heart rate remained constant during the procedure allowing valid comparison of the load-dependent parameters studied. Whilst it would also have been statistically preferable to enroll a larger number of patients, ethical and practical considerations limited numbers to those specified in the power calculation.

In the demand ischemia protocol it would have been preferable to perform prospectively recruited saline and glibenclamide only DSEs to control comprehensively. This was not possible for both ethical and practical reasons. We instead relied upon our historic control data using the same protocol, which has demonstrated that GLP-1 augments myocardial performance during demand ischemia [16].

## Conclusion

The cardioprotective effect of GLP-1 (7–36) amide in non-lethal IR ventricular injury does not appear to be mediated through an mK-ATP dependent pathway. Changes in myocyte substrate utilization and workload after GLP-1 also fail to explain the protection observed. Future mechanistic studies need to consider alternative mechanisms to explain the beneficial effect of GLP-1.

## Additional file

10.1186/s12933-016-0416-3 Additional material.

## Abbreviations

AMI: acute myocardial infarction
BL1: baseline 1
BL2: baseline 2
BO1: balloon occlusion 1
BO2: balloon occlusion 2
DSE: dobutamine stress echocardiogram(s)
EDP: end diastolic pressure
EF: ejection fraction
GLP-1: glucagon-like peptide 1 (7–36) amide
ICC: intra-class correlation coefficient
IPC: ischemic preconditioning
IR: ischemia-reperfusion
LAD: left anterior descending artery
LCX: left circumflex artery
LV: left ventricle
MASV: mitral annular systolic velocity
mK-ATP: mitochondrial K-ATP channel
PCI: percutaneous coronary intervention
RCA: right coronary artery
SV: stroke volume
