# Supplementary material for: Glucagon-like peptide-1 derived cardioprotection does not utilize a KATP-channel dependent pathway: mechanistic insights from human supply and demand ischemia studies
Source: Cardiovasc Diabetol. 2016 Jul 19;15:99. doi: 10.1186/s12933-016-0416-3 (PMC4950774; doi:10.1186/s12933-016-0416-3)
Supplement: Supplementary file 1 — 10.1186/s12933-016-0416-3 Additional material. [file 12933_2016_416_MOESM1_ESM.docx]

**Supplementary Material for Online Data Supplement**

# Glucagon-like Peptide-1 derived cardioprotection does not utilize a KATP-channel dependent pathway: mechanistic insights from human supply and demand ischemia studies

Joel P. Giblett BM BSc MRCP*^†^, Richard Axell MSc^‡^, Paul White PhD^‡^, Sophie Clarke BSc^†^, Liam McCormick MD MRCP*, Philip Read MD MRCP*, Johannes Reinhold MBBS MRCP^†^, Adam Brown PhD MRCP*^†^, Michael O’Sullivan MA PhD FRCP*, Nick E. J. West MA MD FRCP*, David P. Dutka MD FRCP^†^, Stephen P. Hoole MA DM FRCP *

*Department of Interventional Cardiology, Papworth Hospital, Cambridge, UK

†Department of Cardiovascular Medicine, University of Cambridge, UK

‡Department of Clinical Engineering, Addenbrooke’s Hospital, Cambridge, UK

Correspondence:

Dr Stephen Hoole

Department of Interventional Cardiology

Papworth Hospital

Papworth Everard

Cambridge

CB23 3RE, UK

Tel: +44 1480 364119

Fax: +44 1480 364799

Email: [stephen.hoole@papworth.nhs.uk](mailto:stephen.hoole@papworth.nhs.uk)

**Detailed Methods**

**Calibration of the conductance catheter (supply protocol)**

The 8-electrode conductance catheter was placed along the longitudinal axis of the LV using fluoroscopic guidance. The tip of the catheter was located in the apex of the LV. The conductance catheter was connected to a signal conditioning unit and measures time-varying conductance (G(t)) as the sum of the conductance between the central 6 electrodes when a 20 Hz current is applied to the proximal and distal electrodes. The methodology for calibration was described by Baan et al^1^. Time-varying volume, V(t), was calculated according to the formula:

V(t) = (1/α) × (L^2^/σ) × G(t) – Vc

α is the ratio of conductance-derived volume to true ventricular volume determined by Fick, L is the inter-electrode distance, σ is the specific conductivity of blood that is measured at the beginning of the case, and Vc is a volume correction to account for the conductance of structures parallel to the blood in the ventricular cavity. The Vc was calculated from parallel conductance (Gp) as follows:

Vc = (1/α) × (L^2^/σ) × Gp.

Gp was measured by injecting 5ml hypertonic saline (10%) through a multipurpose catheter inserted into the pulmonary artery during a mid-expiration breath hold.

**Figure S1**

**
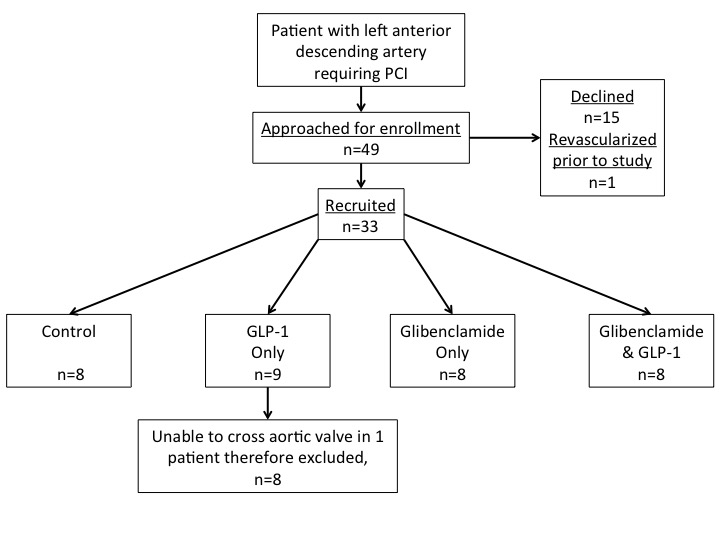
**

Consort diagram to show recruitment and participation in supply ischemia protocol.

## Table S1 – Demographics of supply ischemia protocol

|  | | Control | GLP-1 | GLP-1 Glibenclamide | Glibenclamide | p-value |
| --- | --- | --- | --- | --- | --- | --- |
| Age, years, median [IQR] | | 58 [55.5-63.5] | 62.5 [59.5-78.5] | 63 [57.5-77] | 67 [56-71] | 0.26 |
| Male Sex, n (%) | | 7 (87.5) | 7 (87.5) | 7 (87.5) | 7 (87.5) | 1.00 |
| BMI, Kg.m^-2^ | | 32.2±2.9 | 31.2±5.4 | 28.6±2.1 | 33.1±5.2 | 0.25 |
| Previous MI, n (%) | | 3 (37.5) | 2 (25) | 1 (12.5) | 2 (25) | 0.72 |
| Active or Ex Smoker, n (%) | | 4 (50) | 6 (75) | 3 (37.5) | 4 (50) | 0.50 |
| Hypertension, n (%) | | 3 (37.5) | 4 (50) | 4 (50) | 2 (25) | 0.70 |
| Medication | |  |  |  |  |  |
|  | Beta Blocker, n (%) | 7 (87.5) | 7 (87.5) | 7 (87.5) | 5 (62.5) | 0.48 |
|  | ACE inhibitor, n (%) | 4 (50) | 4 (50) | 1 (12.5) | 4 (50) | 0.54 |
|  | Ca Channel Blocker, n (%) | 2 (25) | 2 (25) | 2 (25) | 2 (12.5) | 0.91 |
|  | Long acting nitrate, n (%) | 2 (25) | 3 (37.5) | 4 (50) | 3 (37.5) | 0.79 |
| Baseline Bloods | |  |  |  |  |  |
|  | Hemoglobin, g/L | 145±8.1 | 128±23.5 | 143±11.6 | 138±9.2 | 0.17 |
|  | Creatinine, μmol/l | 94.8±29.0 | 97.0±23.4 | 86.2±35.4 | 89.6±19.9 | 0.97 |
|  | Cholesterol, mmol/L | 3.61±0.8 | 4.20±0.90 | 3.99±1.1 | 4.16±1.5 | 0.73 |
|  | Blood Glucose, mmol/l | 5.1±0.3 | 5.0±0.3 | 5.3±0.3 | 5.5±0.3 | 0.87 |

## *p-value for inter-group comparison

# Table S2 – Baseline left ventricular hemodynamic data in supply ischemia protocol

|  | Control, | GLP-1 Only | Glibenclamide Only | GLP-1 Glibenclamide | p-value* |
| --- | --- | --- | --- | --- | --- |
| Heart Rate, BPM | 58±8 | 70±14 | 61±8 | 61±8 | 0.13 |
| Mean Arterial Pressure, mmHg | 93±9 | 88±17 | 88±12 | 88±17 | 0.31 |
| LV EDP, mmHg | 14.3±2.5 | 19.7±7.6 | 16.8±2.9 | 13.8±2.9 | 0.27 |
| Stroke Volume, ml | 74.7±19.8 | 75.2±21.2 | 79.5±23.1 | 87.1±30.1 | 0.69 |
| Ejection Fraction, % | 60.6±8.7 | 60.8±7.26 | 57.3±8.4 | 64.2±4.7 | 0.51 |
| Cardiac Output (Fick), L/min | 4.6±0.9 | 5.1±1.3 | 5.5±1.1 | 5.6±0.7 | 0.24 |
| dP/dt_max_, mmHg/s | 1646±259 | 1465±313 | 1457±515 | 1486±306 | 0.77 |
| dP/dt_min_, mmHg/s | -2134±315 | -2051±380 | -1828±719 | -1853±417 | 0.53 |
| Tau, ms | 50.0±11.2 | 58.9±10.0 | 55.4±10.0 | 48.1±5.9 | 0.14 |

# *p-value for 4 way inter-group comparison

## Table S3 – Demographics of demand ischemia protocol

| Age, median [IQR] | | 65.5 [57.5-71] |
| --- | --- | --- |
| Male Sex, n (%) | | 9 (90) |
| BMI, Kg/m^2^ | | 30.7±4.89 |
| Previous MI, n (%) | | 1 (10) |
| Active or Ex Smoker, n (%) | | 6 (60) |
| Hypertension, n (%) | | 4 (40) |
| Hypercholesterolemia, n (%) | | 4 (40) |
| Planned Revasc. (CABG), n (%) | | 5 (50) |
| Medication | |  |
|  | Beta Blocker, n (%) | 6 (60) |
|  | ACE inhibitor, n (%) | 6 (60) |
|  | Ca Channel Blocker, n (%) | 0 (0) |
|  | Long acting nitrate, n (%) | 4 (40) |
| Baseline Bloods | |  |
|  | Haemoglobin, g/l | 144.5±11.6 |
|  | Creatinine, μmol/l | 77.2±17.2 |
|  | Cholesterol, mmol/l | 3.79±0.66 |

# Table S4 – Hemodynamic changes during demand ischemia protocol

|  | GLP-1 Only | GLP-1 Glibenclamide | p-value |
| --- | --- | --- | --- |
| Heart Rate (Baseline), BPM | 60±10.5 | 64±8.6 | 0.23 |
| Heart Rate (Pre DSE), BPM | 58±7.6 | 57±11.2 | 0.83 |
| Heart Rate (Peak Stress), BPM | 115±11.5 | 119±14.2 | 0.12 |
| Heart Rate (Recovery), BPM | 71±11.9 | 67±9.1 | 0.34 |
| Systolic Blood Pressure (Peak Stress), mmHg | 159±35 | 157±32 | 0.82 |
| Diastolic Blood Pressure (Peak Stress), mmHg | 75±10 | 77±9 | 0.62 |
| Rate Pressure Product (Peak Stress), mmHg | 18059±2562 | 18675±3647 | 0.59 |

# Table S5 – Metabolic changes during demand ischemia protocol

|  | | Baseline | | | | | Pre GLP-1 | | | | | | Pre-DSE | | | | Peak Stress | | | | | | 30-minute recovery | | |
| --- | --- | --- | --- | --- | --- | --- | --- | --- | --- | --- | --- | --- | --- | --- | --- | --- | --- | --- | --- | --- | --- | --- | --- | --- | --- |
| GLP-1 (7-36) amide, pg/ml | | | | |  | | | | |  | | | | |  | | | | |  | | | | | |
|  | GLP-1 Only | 2.0±2.6 | | | | | 2.2±2.9 | | | | | | 32.4±32.4 | | | | 103.7±57.5 | | | | | | 110.6±46.6 | | |
|  | GLP-1 Glibenclamide | 1.2±1.3 | | | | | 1.1±0.65 | | | | | | 24.2±20.3 | | | | 102.6±26.2 | | | | | | 115.8±59.4 | | |
|  | p-value | 0.94 | | | | | 0.67 | | | | | | 0.15 | | | | 0.96 | | | | | | 0.25 | | |
| Insulin, pmol/L | |  | | |  | | | | |  | | | | |  | | | | |  | | | | | |
|  | GLP-1 Only | 68±28 | | | | | | 118±65 | | | | | | 191±173 | | | | 732±679 | | | | | | 230±117 | |
|  | GLP-1 Glibenclamide | 50±19 | | | | | | 141±78 | | | | | | 379±292 | | | | 1577±1174 | | | | | | 828±527 | |
|  | p-value | 0.10 | | | | | | 0.86 | | | | | | 0.22 | | | | 0.04 | | | | | | 0.07 | |
| Glucose, mmol/l | |  | | | |  | | | | |  | | | |  | | | | | |  | | | | |
|  | GLP-1 Only | 4.9±0.11 | | | | | | 7.3±1.9 | | | | | | 6.4±0.7 | | | | 6.6±1.1 | | | | | | 5.7±1.0 | |
|  | GLP-1 Glibenclamide | 4.9±0.16 | | | | | | 7.3±1.7 | | | | | | 6.0±0.6 | | | | 5.6±0.9 | | | | | | 4.7±0.9 | |
|  | p-value | 0.82 | | | | | | 0.98 | | | | | | 0.12 | | | | 0.06 | | | | | | 0.02 | |
| Free Fatty Acids, μmol/L | | | |  | | | | |  | | |  | | | |  | | |  | | |  | | |  |
|  | GLP-1 Only | 332±126 | | | | | 223±95 | | | | | | 195±74 | | | | 687±402 | | | | | | 243±179 | | |
|  | GLP-1 Glibenclamide | 374±66 | | | | | 188±35 | | | | | | 144±45 | | | | 435±210 | | | | | | 103±79 | | |
|  | p-value | 0.07 | | | | | 0.80 | | | | | | 0.46 | | | | 0.14 | | | | | | 0.31 | | |
| Dextrose Infusion, mg.Kg^-1^.min^-1^ | | |  |  |  | | | |  | | |  | | | |  | | |  | | |  | | |  |
|  | Glibenclamide Only | 1.5±0 | | | | | 1.5±0 | | | | | | 1.5±0 | | | | 1.5±0 | | | | | | 1.5±0 | | |
|  | GLP-1 Glibenclamide | 1.5±0 | | | | | 1.5±0 | | | | | | 1.5±0 | | | | 1.5±0 | | | | | | 1.9±0.6 | | |
|  | p-value | - | | | | | - | | | | | | - | | | | - | | | | | | 0.17 | | |

**Table S6 – Changes in systolic function within all myocardial segments in demand ischemia protocol**

|  | | | GLP-1 Only | GLP-1 Glibenclamide | p-value |
| --- | --- | --- | --- | --- | --- |
| Peak Systolic Velocity (Vs), cm/s | |  |  |  |  |
|  | Baseline | | 3.97±2.05 | 4.09±2.21 | 0.45 |
|  | Peak Stress | | 9.97±3.07 | 9.69±2.87 | 0.17 |
|  | Recovery | | 4.26±2.02 | 4.26±1.60 | 0.58 |
| Strain Index (SI), % | |  |  |  |  |
|  | Baseline | | -15.3±5.79 | -15.2±5.24 | 0.91 |
|  | Peak Stress | | -15.3±5.66 | -15.6±5.79 | 0.65 |
|  | Recovery | | -15.0±5.27 | -15.3±6.06 | 0.59 |
| Strain Rate Index (SRI), s^-1^ | |  |  |  |  |
|  | Baseline | | -1.18±0.40 | -1.22±0.40 | 0.38 |
|  | Peak Stress | | -2.72±1.11 | -2.70±0.91 | 0.81 |
|  | Recovery | | -1.26±0.52 | -1.25±0.53 | 0.86 |

**Supplementary References**

1. Baan J, van der Velde ET, de Bruin HG, Smeenk GJ, Koops J, van Dijk AD, Temmerman D, Senden J, Buis B. Continuous measurement of left ventricular volume in animals and humans by conductance catheter. Circulation 1984;**70**(5):812-23.
